# Microwave Ablation Monitoring Using Thermoacoustic and Ultrasound Tomography

**DOI:** 10.1109/jmw.2025.3612329

**Published:** 2025-10-10

**Authors:** DAVID C. GARRETT, YOUSUF ABORAHAMA, JINHUA XU, GENG KU, LIHONG V. WANG

**Affiliations:** Caltech Optical Imaging Laboratory, Andrew and Peggy Cherng Department of Medical Engineering, Department of Electrical Engineering, California Institute of Technology, Pasadena, CA 91125 USA

**Keywords:** Microwave ablation, ultrasonic imaging, medical diagnostic imaging, photoacoustic imaging, chirp modulation

## Abstract

Microwave ablation is an established minimally invasive technique that induces thermal necrosis in centimeter-scale tumors in organs such as the liver and kidney. However, the efficacy of clinical protocols is challenged by patient-specific and tissue-specific variations that may require personalized parameters to achieve optimal therapeutic outcomes. To address these limitations, we introduce an imaging technique employing modulated microwave signals that maintain peak and average powers comparable to those of existing clinical systems (~100–200 W). This modulation introduces time-varying tissue heating, generating detectable thermoacoustic signals that are used to reconstruct images proportional to the spatial heat deposition. When combined with thermal models of the tissue, we estimate the spatial temperature profile and ablation status over time. We validate this approach in ex vivo bovine liver compared against ultrasound tomography and tissue dissection. This approach may enable closed-loop monitoring and adaptive control of ablation procedures to optimize lesion coverage while sparing surrounding healthy tissue.

## I. INTRODUCTION

Microwave ablation (MWA) has emerged as a preferred minimally invasive approach for treating cancerous tissues, offering reduced recovery times and lower complication risks compared to traditional surgical methods [1], [2]. Unlike radiofrequency ablation, which is limited by tissue charring and impedance changes that restrict energy delivery [3], MWA generates larger, more uniform heating zones through a needle-based radiating element [4], [5] typically operating at 915 MHz or 2.45 GHz. However, the success of MWA treatments relies on a delicate balance between comprehensive tumor cell elimination and the preservation of surrounding healthy tissue. Currently, clinical MWA protocols use standardized ablation parameters established from ex vivo bovine liver experiments, with microwave power (10–200 W) and duration selected according to the desired ablation size. However, patient- and tissue-specific variations may result in suboptimal ablation [6], [7], for instance, due to differences in tumor type or proximity to blood vessels. Although imaging modalities like CT and MRI can effectively evaluate the ablation zone post-procedure, neither of them offers practical methods of real-time assessment during treatment [8], [9]. Contrast-enhanced ultrasound can provide intraoperative visualization, but its usefulness is limited by operator dependence, shallow imaging windows, and the short lifespan of microbubbles in circulation (several minutes) [10]. This gap highlights the need for imaging technology that provides immediate and accurate intraoperative feedback during MWA procedures.

Thermoacoustic (TA) tomography has recently emerged as a promising method to track tissue changes during MWA. Thermoacoustic signals arise from time-varying heating rates in tissues due to transient microwave absorption. These MHz-scale acoustic signals are collected by acoustic detectors and are used to reconstruct images of the microwave absorption profile. TA systems have been more commonly developed for medical imaging, generally using high-power (~kW to MW) sources with sub-microsecond pulse duration. Two approaches have been investigated for TA monitoring of MWA: using an independent TA system with a separate antenna to image tissue property changes [11], [12], or simultaneously ablating and generating TA signals using a pulsed microwave source coupled to the ablation probe [13], [14]. Using a TA imaging system during MWA poses several practical challenges, including achieving sufficient imaging depth for clinically relevant tissue volumes, shielding other devices from the strong microwave signals, and positioning a separate antenna to illuminate the ablation region. Performing TA imaging using signals generated from the ablation probe is advantageous in isolating changes from the ablation region. Previous approaches using pulsed sources, however, require custom probe designs to avoid dielectric breakdown at high peak microwave power, have low average microwave power (<10 W), and require specialized microwave sources like pulsed magnetrons or klystrons. Demonstrations to date have been limited to single-element acoustic receivers, where the extracted parameters of time of arrival and amplitude may not be robust against patient positioning and tissue shrinkage. Furthermore, high-power microwave sources may not be compatible with the sensitive electronics present in clinical settings.

Here, we propose an approach for TA monitoring of MWA using similar peak (~190 W) and average (100 W) microwave powers as existing commercial systems. By modulating a 2.45 GHz continuous-wave (CW) microwave source at ultrasound-scale frequencies (~MHz), TA signals are generated without altering the heat deposition while providing spatial information on the deposited microwave energy. We perform this using a commercial microwave power amplifier and MWA probe. The TA signals are detected using a human-scale 512-element acoustic detector array, and they are used to obtain TA images of the ablated region with temporal resolution of 1 s throughout procedures of several minutes. We demonstrate this technique in ex vivo bovine liver samples. We calibrate our TA images to a heating function by considering the temperature-dependent thermal expansion coefficient in tissues like liver, which we relate to an observed initial rise in TA amplitude during the first few seconds of the procedure. Ultrasound tomography (UST) is also used to image the location of the needle with respect to tissue features and to observe tissue changes due to ablation. This approach could be readily adapted to commercial systems and may allow for more precise MWA treatment.

## II. METHODS

**FIGURE 1.**
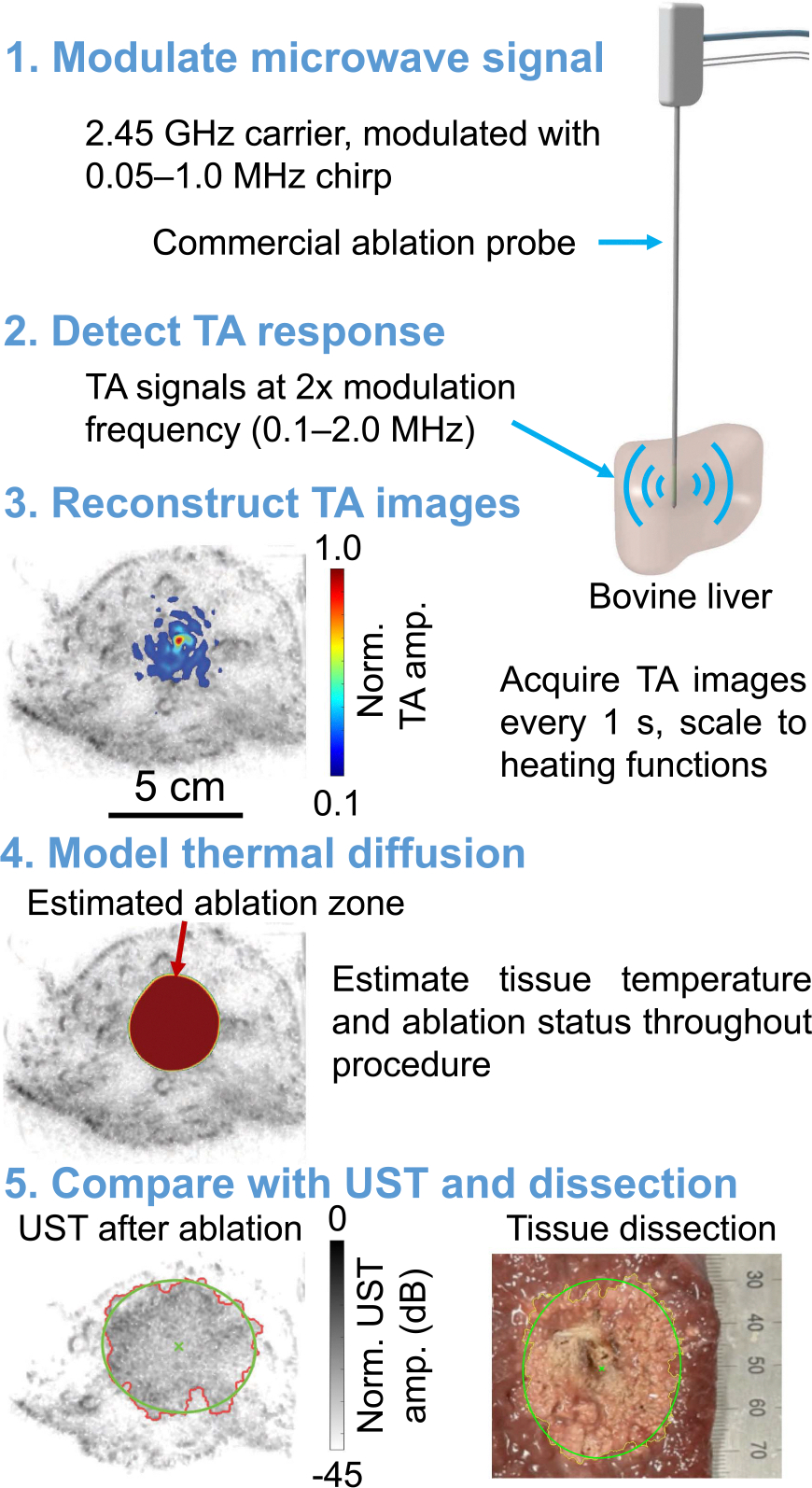
Overview of the methodology for thermoacoustic (TA) ablation monitoring. UST: ultrasound tomography.

Our methodology is outlined in Fig. 1. First, we excite the ablation probe using a modulated microwave source to generate detectable TA signals. We employ a linear chirp to enhance the signal-to-noise ratio (SNR) of broadband detected TA profiles given a limited peak microwave power. Due to their quadratic relation to the applied electric field, the resulting TA signals arise at twice the modulation frequencies. These TA signals are captured using a human-scale, 512-element ultrasonic ring array. The recorded signals are deconvolved with the expected chirp and are then used to reconstruct TA images throughout the ablation procedure. We then calibrate these images to estimate the heating function in absolute units, which serves as the source term in a thermal diffusion model incorporating temperature-dependent tissue properties. Finally, we estimate the ablation zone using the Arrhenius damage integral and compare it with photographs of the dissected tissue samples and with UST images captured before and after ablation.

### A. THERMOACOUSTIC SIGNAL GENERATION

During time-varying microwave-induced heating Q(r,t), the resulting TA pressure wave equation is [15]:

(1) $$\left(\nabla^{2}-\frac{1}{v_{s}^{2}(r)}\frac{\partial^{2}}{\partial t^{2}}\right)p(r,\;t,\;\tau )=-\frac{\beta (r,\;\tau )}{C_{p}(r,\;\tau )}\frac{\partial Q(r,\;t,\;\tau )}{\partial t}$$

where p(r,t,τ) is the TA pressure, $v_{s}$ is the tissue speed of sound, β is the coefficient of thermal expansion, and $C_{p}$ is the specific heat capacity at constant pressure. We use two time scales: t denotes the fast (~microseconds) variation introduced by the modulated microwave absorption, and τ denotes the slow (~seconds) variation due to temperature change and tissue ablation. Note that (1) is valid under thermal confinement, which is met with fast-time variation in the source term. The heating function in tissue due to narrowband microwave absorption is found as

(2) $$Q(r,\;t,\;\tau )=\sigma_{eff}(r,\;\tau )E^{2}(r,\;t,\;\tau ),\;$$

where E(r,t,τ) is the instantaneous scalar electric field amplitude. The static conductivity $\sigma_{s}$ and dielectric loss ${\varepsilon_{r}}^{\prime \prime }$ are combined into a single term $\sigma_{\text{eff}}=\sigma_{s}+2\pi f_{c}\varepsilon_{0}{\varepsilon_{r}}^{\prime \prime }$, where $f_{c}$ is the microwave carrier frequency. We assume that $\sigma_{\text{eff}}$ is approximately constant over a narrow modulation frequency $f_{m}\ll f_{c}$, but that it may vary over ~seconds due to changing temperature and ablation status as water content and protein structures are altered. Magnetic losses are considered negligible in endogenous tissue ($\mu_{r}^{\prime \prime }∼0$).

To generate detectable TA signals, we modulate the microwave carrier frequency $f_{c}=2.45\;GHz$ at acoustic-scale frequencies using double-sideband suppressed carrier (DSB-SC) modulation. To improve the SNR with a limited peak microwave power, we modulate the microwave carrier signal using a chirp. A similar approach has been used for TA imaging at nontherapeutic power levels [16]. The linear chirp has time-varying frequency $f_{m}(t)=f_{r}t+f_{0}$, where $f_{r}=\left(f_{1}-f_{0}\right)/t_{c}$ is the linear chirp rate, $f_{0}=0.05\;MHz$ is the lower frequency, $f_{1}=1.0\;MHz$ is the upper frequency, and $t_{c}=400\;\mu s$ is the chirp duration. These parameters are chosen based on the bandwidth of our acoustic receivers and the maximal recording time of our data acquisition hardware. The modulating chirp signal is

(3) $$x(t)=sin\left[2\pi \left(\frac{f_{r}}{2}t^{2}+f_{0}t\right)\right],\;0\leq t\leq t_{c}.$$

The temporal variation of the electric field is therefore:

(4) $$E(r,\;t,\;\tau )=E_{0}(r,\;\tau )x(t)sin\left(2\pi f_{c}t\right).$$

We assume that the electric field amplitude $E_{0}(r,\;\tau )$ is constant across the narrow modulation bandwidth but may vary slowly during the ablation procedure. Since the heating rate is proportional to the squared electric field,

(5) $$\begin{array}{l} Q(r,\;t,\;\tau )\;=\frac{1}{4}\sigma_{eff}(r,\;\tau )E_{0}^{2}(r,\;\tau )g(t),\;\\ \;g(t)\;=\left[1-cos\left[4\pi \left(\frac{f_{r}}{2}t^{2}+f_{0}t\right)\right]+A_{MW}(t)\right],\;\\ \;0\leq t\leq t_{c},\; \end{array}$$

where $A_{MW}(t)$ are terms with frequencies of $f_{c}\pm f_{m}$ and $2f_{c}$. Since $f_{c}$ is in the GHz range, TA signals corresponding to these terms would quickly attenuate and fall outside of our detection band, so we do not consider them in our analysis. By differentiating (5) and substituting into the right-hand side of (1), the resulting TA source term is

(6) $$p_{s}(r,\;t,\;\tau )=-\pi \frac{\beta (r,\;\tau )}{C_{p}(r,\;\tau )}Q_{0}(r,\;\tau )f_{m}(t)x_{2f}(t),\;$$

where $Q_{0}(r,\;\tau )=\sigma_{\text{eff}}(r,\;\tau )E_{0}^{2}(r,\;\tau )$ is the average heating function which is expected to vary on the order of seconds as the tissue becomes ablated, and $x_{2f}(t)=sin\left[2\pi f_{r}t^{2}+4\pi f_{0}t\right],\;0\leq t\leq t_{c}$ is the linear chirp at twice the modulation frequency (0.1–2.0 MHz). Note that the TA pressure is directly proportional to the average heating function $Q_{0}(r,\;\tau )$ in (6) but scaled through $-\pi \left(\frac{\beta (r,\;\tau )}{C_{p}(r,\;\tau )}\right)f_{m}(t)$. Importantly, the recovery of $Q_{0}(r,\;\tau )$ from the TA signals does not require knowledge of $\sigma_{\text{eff}}(r,\;\tau )$ or $E_{0}(r,\;\tau )$, which are difficult to estimate in tissues.

**FIGURE 2.**
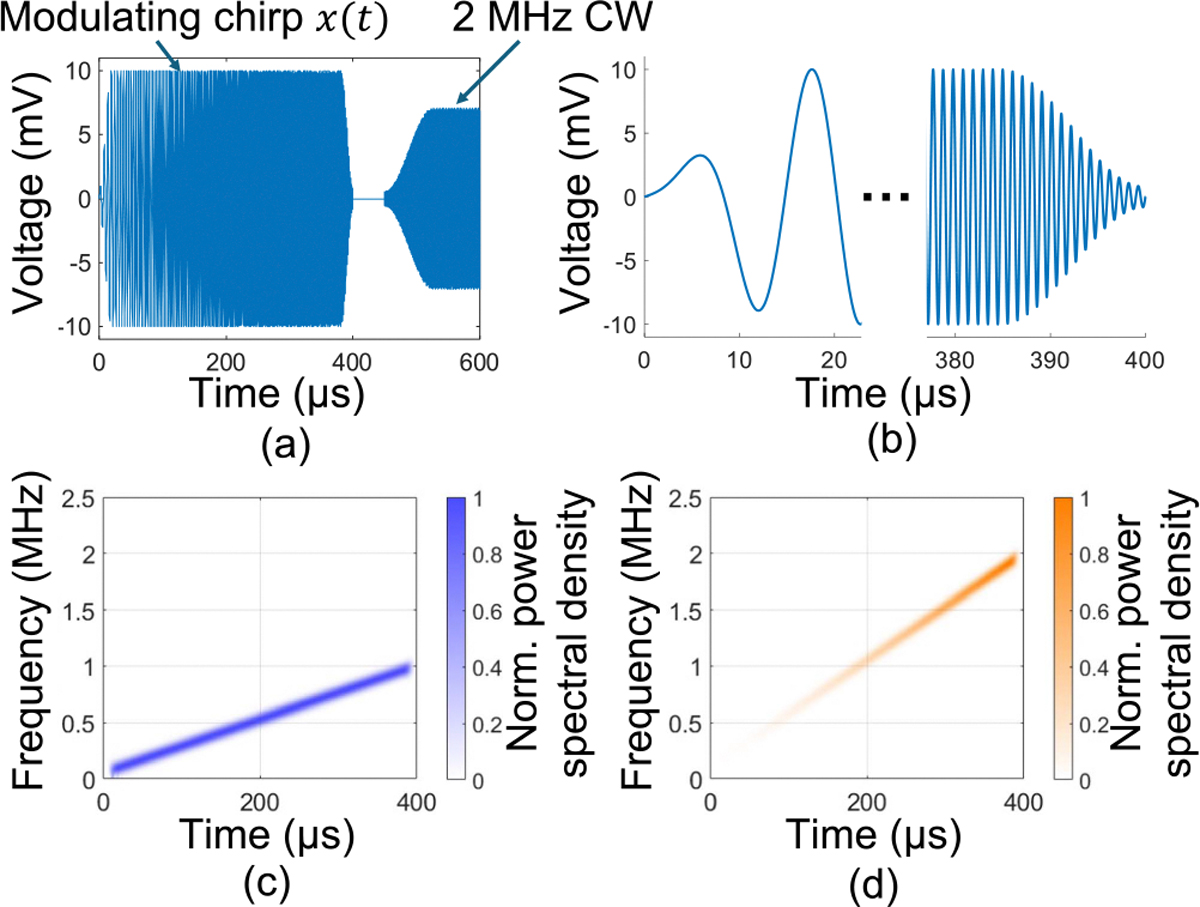
Modulating waveform and expected TA source signal structure. (a) Time domain waveform used to modulate the 2.45 GHz microwave source. The 400 *μ*s chirp is followed by a ~9.5 ms CW signal to maintain microwave energy deposition during a 10 ms period. (b) Hamming windows are applied to the beginning and end of the chirp. (c) Spectrogram of the modulating chirp spanning 0.05–1.0 MHz. (d) Spectrogram of the expected TA signal source term, consisting of a ramp-weighted chirp spanning 0.1–2.0 MHz.

To maintain average microwave power comparable to clinical systems (100 W), after the chirp we apply a 9.5 ms single-tone modulating signal at half the power of the chirp. The modulation waveforms are shown in Fig. 2, where we apply Hamming windows on the waveform edges to reduce sidelobes after deconvolution.

We then recover a pulse-like response $\chi_{s,i}(t)$ from the recorded TA signals $x_{TA,i}(t)$ on each detector i using Wiener deconvolution with the expected response $r(t)=\left(f_{r}t+f_{0}\right)x_{2f}(t)$:

(7) $$\chi_{s,i}(t)=F^{-1}\left\{\frac{R^{*}(f)}{|R(f){|}^{2}+\lambda }X_{TA,i}(f)\right\},$$

where $F^{-1}$ denotes the inverse Fourier transform, R(f) and $X_{TA,i}(f)$ are the Fourier transforms of r(t) and $x_{TA,i}(t)$, respectively, and λ is a noise-to-signal parameter (set to 0.1) that regularizes the inversion. Multiplying this filter with the spectrum of the recorded signals suppresses noise amplification while compensating for the system response. The pulse-like responses $\chi_{s,i}(t)$ from each channel are then used to reconstruct images of the estimated heating function. Compared to a microwave pulse with similar peak power, a linear chirp results in an expected SNR improvement of $∼\sqrt{t_{c}\cdot \left(f_{1}-f_{0}\right)}$ [17].

### B. SYSTEM HARDWARE

**FIGURE 3.**
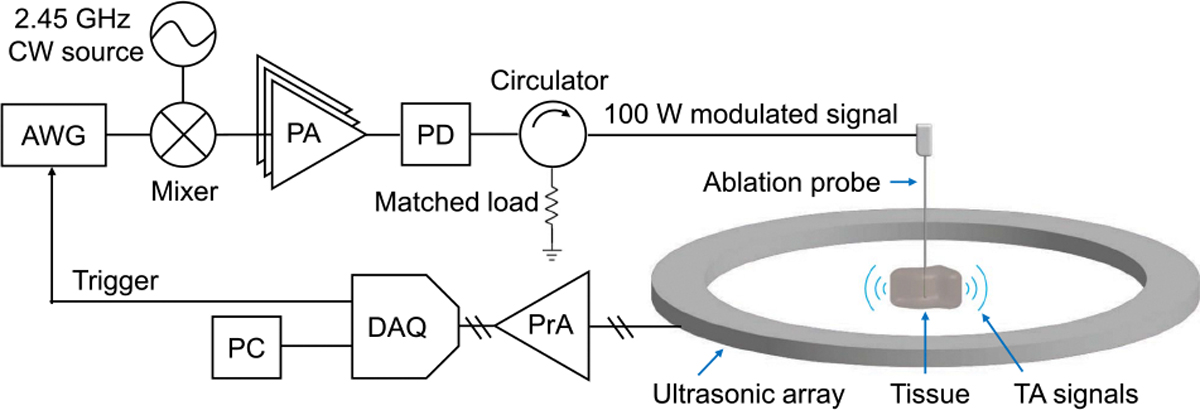
System hardware for thermoacoustic ablation monitoring. AWG: arbitrary waveform generator. PA: power amplifier (in three stages). PD: power detector. PrA: preamplifiers. DAQ: data acquisition module. PC: personal computer. CW: continuous-wave.

We show the system hardware in Fig. 3. Note that a similar microwave power amplifier is used as in clinical systems [18], and that the microwave modulation only requires a modulating source (from a low-cost arbitrary waveform generator) and a mixer. The chirp waveform x(t) is first programmed in an arbitrary waveform generator (AWG, Siglent SDG2042) and coupled to one input of a mixer (Analog Devices DC1983A) with 20 mV_pp_ maximum amplitude. The other mixer input is fed from a microwave source (LibreVNA) providing a 2.45 GHz CW signal at −1 dBm. The modulated signal is then amplified in two stages (Mini-Circuits ZX-60–43S+ and Mini-Circuits PHA-102+), followed by a high-power amplifier (Mini-Circuits ZHL-2425–250X+). The resulting modulated signal consists primarily of two first-order sidebands in the frequency domain at 2.45 $GHz\;\pm \;f_{m}(t)$. The carrier and higher-order sidebands remain at least 15 dB below the first-order sidebands. We use an in-line power detector (Bird 5012D) to record the peak and average microwave power delivered to the probe. A circulator (Ditom D3C2327N) directs the reflected signals from the probe into a matched load.

We use a 20 cm Covidien Emprint ablation probe, which has a 2.45 GHz operating frequency and supports 100 W average power. The probe is water-cooled using a peristaltic pump during operation (Emprint Ablation Pump). A fiber-optic thermometer (Omega FOM-L201 and FOS-LT-5) is also inserted in the tissue to monitor the temperature. The tissue sample is immersed in water for acoustic coupling to the receiver array. It is mounted on a plastic support plate held by a single stainless-steel post.

**FIGURE 4.**
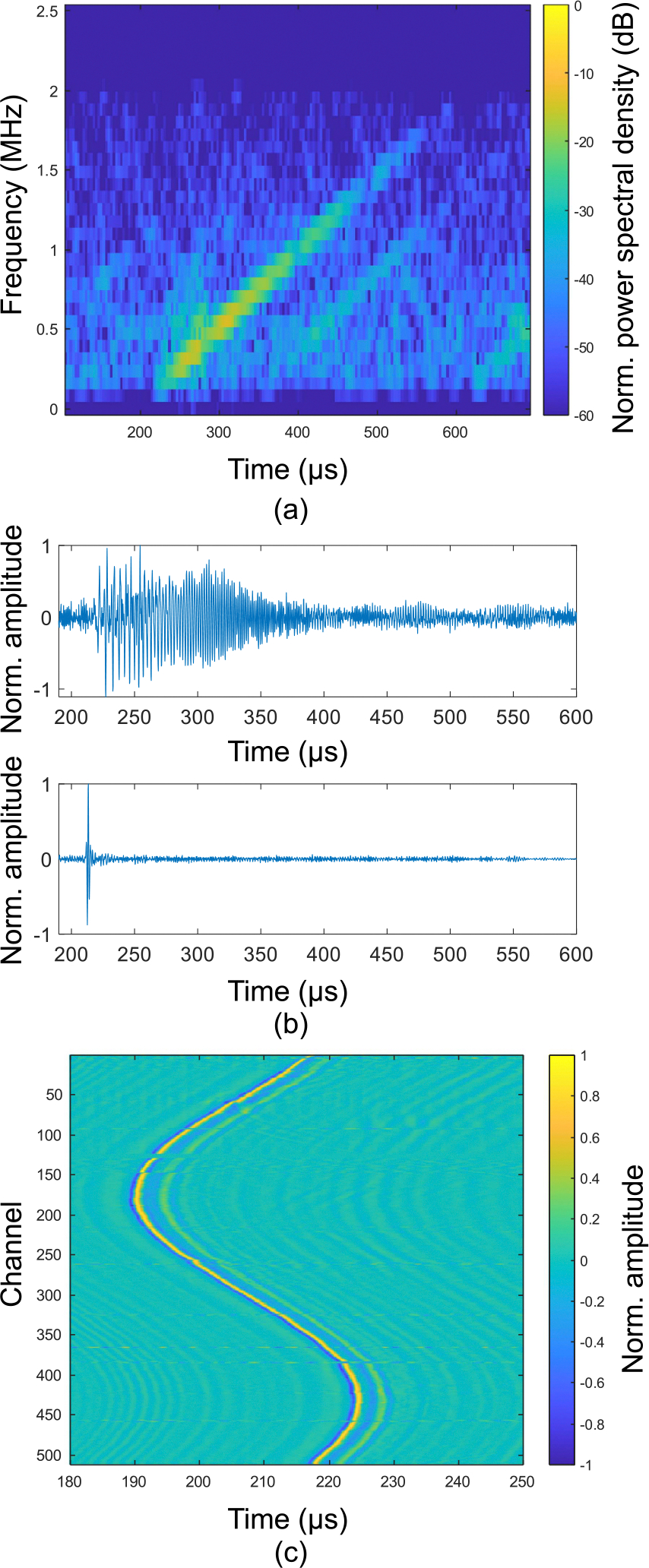
Recorded and deconvolved TA signals for the ablation probe in water. (a) Spectrogram of an example recorded TA channel. The recorded chirp spans the expected range (~0.1–2.0 MHz) covering twice the modulation frequencies (0.05–1.0 MHz). (b) Top: Example recorded TA signal. Bottom: example TA signal after deconvolution with the expected scaled chirp response. (c) Deconvolved response over all array elements. These signals are then used for image reconstruction.

The ablation probe is inserted into tissue, and the generated TA signals are launched in water immersion. We detect the TA signals using the custom 512 element, 60 cm diameter acoustic receiver array with 1 MHz center frequency described in [19]. Each channel is preamplified by 15 dB and digitized in parallel at 5 MSPS (Photosound Legion) using 51 dB gain. We acquire TA signals at a 100 Hz repetition rate. On each trigger, the 400 *μ*s chirp-modulated signal is delivered to the ablation probe. We average and store the detected signals every 100 shots, corresponding to 1-second acquisitions. Before and after microwave ablation, we also image the sample using UST with the approach described in [19]. We show an example spectrogram and deconvolved TA response from the ablation probe immersed in water in Fig. 4. Note that the amplitude of the recorded chirp also depends on the spatial distribution of absorbers. For the broad absorption profiles found here, this results in a low-frequency weighting of the launched TA signals compared to the general source term shown in Fig. 2.

### C. IMAGE RECONSTRUCTION

The detected TA signals are deconvolved with the chirp waveform to recover a pulse-like representation. We then use universal back-projection [20] to recover a TA image proportional to the microwave absorption profile. For each frame, we perform reconstruction using both the deconvolved signals and their Hilbert transform, resulting in TA images $I_{\text{TA,re}}$ and $I_{\text{TA,im.}}$. These images are combined as $I_{\text{TA}}(r,\;\tau )=\sqrt{I_{TA,re}(r,\;\tau {)}^{2}+I_{TA,im}(r,\;\tau {)}^{2}}$, which results in a non-negative image related to the heating function. We apply a 5 cm diameter circular mask centered on the probe to isolate the signal of interest and remove background noise from our thermal model.

We perform TA image reconstruction using a CUDA kernel called from MATLAB, computed using a NVIDIA A100 Tensor Core graphics processing unit (GPU). Reconstruction is performed over a 128 mm × 128 mm grid with 0.25 mm × 0.25 mm pixel size, consisting of 512 × 512 pixels. For each image, the required computational time is approximately 0.3 s. We reconstruct UST images using the method described in [19].

### D. THERMAL MODELLING

We model the thermal dynamics in tissue using the heat diffusion equation

(8) $$\frac{\partial T(r,\;\tau )}{\partial \tau }=\nabla \cdot [\alpha (T(r,\;\tau ))\nabla T(r,\;\tau )]+\frac{Q_{0}(r,\;\tau )}{\rho C_{P}(T(r,\;\tau ))},\;$$

where α is the thermal diffusivity and ρ is the mass density. These properties are expected to vary based on temperature T in °C as [21]

(9) $$\begin{array}{l} \rho C_{P}(T)\approx 3.542+1.79\times 10^{-4}exp(0.233\cdot T)\\ \;\left[MJ\cdot {\;m}^{-3}\cdot {}^{\circ}C^{-1}\right],\\ \alpha (T)\approx 1.55\times 10^{-7}+4.95\times 10^{-16}exp(0.201\cdot T)\\ \;\left[m^{2}\cdot {\;s}^{-1}\right]. \end{array}$$

where MJ denotes units of megajoules. The mass density is assumed constant [22]. We solve (8) numerically in 3D using a grid spacing of 0.25 mm and a time step of 10 ms.

We use the generated TA image series to estimate $Q_{0}(r,\;\tau )$. We first approximate the heating function distribution in the z dimension by considering the radiating region $r_{\text{probe}}\approx 1\;cm$ along the ablation probe. We apply a weighting function $w(z)=\sqrt{r_{\text{probe}}^{2}-z^{2}}$, $|z|<r_{\text{probe}}$. To obtain heating function maps in absolute units, one method is to calibrate the array detection sensitivity where $Q_{0}(r,\;\tau )$ is then found by scaling the pressure in (6). However, acoustic attenuation and scattering in tissue may result in inaccurate absolute pressures recovered deep in tissue. Instead, we take advantage of the variation of β versus temperature near the beginning of the MWA procedure, where temperature increases rapidly before the tissue is ablated. We approximate the coefficient of thermal expansion as linear versus temperature:

(10) $$\beta (T)\approx 3.5\times 10^{-4}+3\times 10^{-6}\left(T-37^{\circ }C\right)\left[{}^{\circ}C^{-1}\right].$$

Since these values are not well characterized in the literature, we use an approximation similar to the temperature-dependence of β in water. We then approximate the TA images to the heating function through a scaling term K. Since for a fixed $Q_{0}(r,\;\tau )$ the TA images would increase in amplitude proportionally to β during heating, we also normalize by the increase in β(T):

(11) $$Q_{0}(r,\;\tau )\approx \frac{K}{\beta (r,\;T)/\beta \left(r,\;T_{0}\right)}w(z)I_{TA}(r,\;\tau ).$$

We compare the TA amplitude rise with the modeled β rise to estimate K. We consider the mean of the TA image $I_{TA}(r,\;\tau )$ in a 5 mm diameter region around the probe tip, denoted $I_{TA,\;\text{avg}}\left(r_{tip},\;\tau \right)$, and the mean of the estimated β over the same region $\beta_{\text{avg}}\left(r_{\mathrm{tip}},\;T\right)$. We normalize both the TA images and β estimates to τ=0, and we estimate K to minimize their difference over the first five seconds of ablation, with τ sampled in discrete 1-s increments:

(12) $$\begin{array}{l} \;\overset{ˆ}{K}\;=\underset{K}{argmin}\sum_{\tau =0}^{\tau =5\;s}\;{\left[I_{n}\left(r_{\mathrm{tip}},\;\tau \right)-\beta_{n}\left(r_{\mathrm{tip}},\;T,\;K\right)\right]}^{2},\\ \;I_{n}\left(r_{\mathrm{tip}},\;\tau \right)\;=\frac{I_{TA,\;\text{avg}}\left(r_{\mathrm{tip}},\;\tau \right)}{I_{TA,\;\text{avg}}\left(r_{\mathrm{tip}},\;0\right)},\\ \beta_{n}\left(r_{\mathrm{tip}},\;T,\;K\right)\;=\frac{\beta_{\text{avg}}\left(r_{\mathrm{tip}},\;T\left(KI_{TA}(r,\;\tau )\right)\right)}{\beta_{\text{avg}}\left(r_{\mathrm{tip}},\;T_{0}\right)}. \end{array}$$

Here, we assume that $Q_{0}(r,\;\tau )$ is constant over the first 5 s, which is supported by the relative consistency of $\sigma_{\text{eff}}(r,\;\tau )$ and the relative permittivity of liver tissue up to ~50 °C [23]. $T\left(KI_{TA}(r,\;\tau )\right)$ numerically solves (8) using the heating function from (11) with scaling factor K.

From the modeled temperature in Kelvin $T_{K}(r,\;\tau )$ throughout the entire MWA procedure, we then estimate the tissue ablation status Ω(r,τ) using the Arrhenius integral [24] as

(13) $$\Omega \left(r,\;\tau \right)=A\int_{0}^{\tau }\exp\left(\frac{-E_{a}}{RT_{K}\left(r,\;\tau^{\prime }\right)}\right)d\tau^{\prime }.$$

A tissue region is considered ablated if Ω>1, where approximately 63% of cells are likely to be ablated. We use typical values [25] for the frequency factor $A=7.39\times 10^{39}{\;s}^{-1}$ and the activation energy $E_{a}=2.577\times 10^{5}\;J/mol$. R=8.314J/(mol⋅K) is the universal gas constant. We model the temperature distribution over the entire ablation procedure and 60 s after microwave exposure. We treat β for ablated tissue as $3\times 10^{-4}{}^{\circ}C^{-1}$.

## III. RESULTS

**FIGURE 5.**
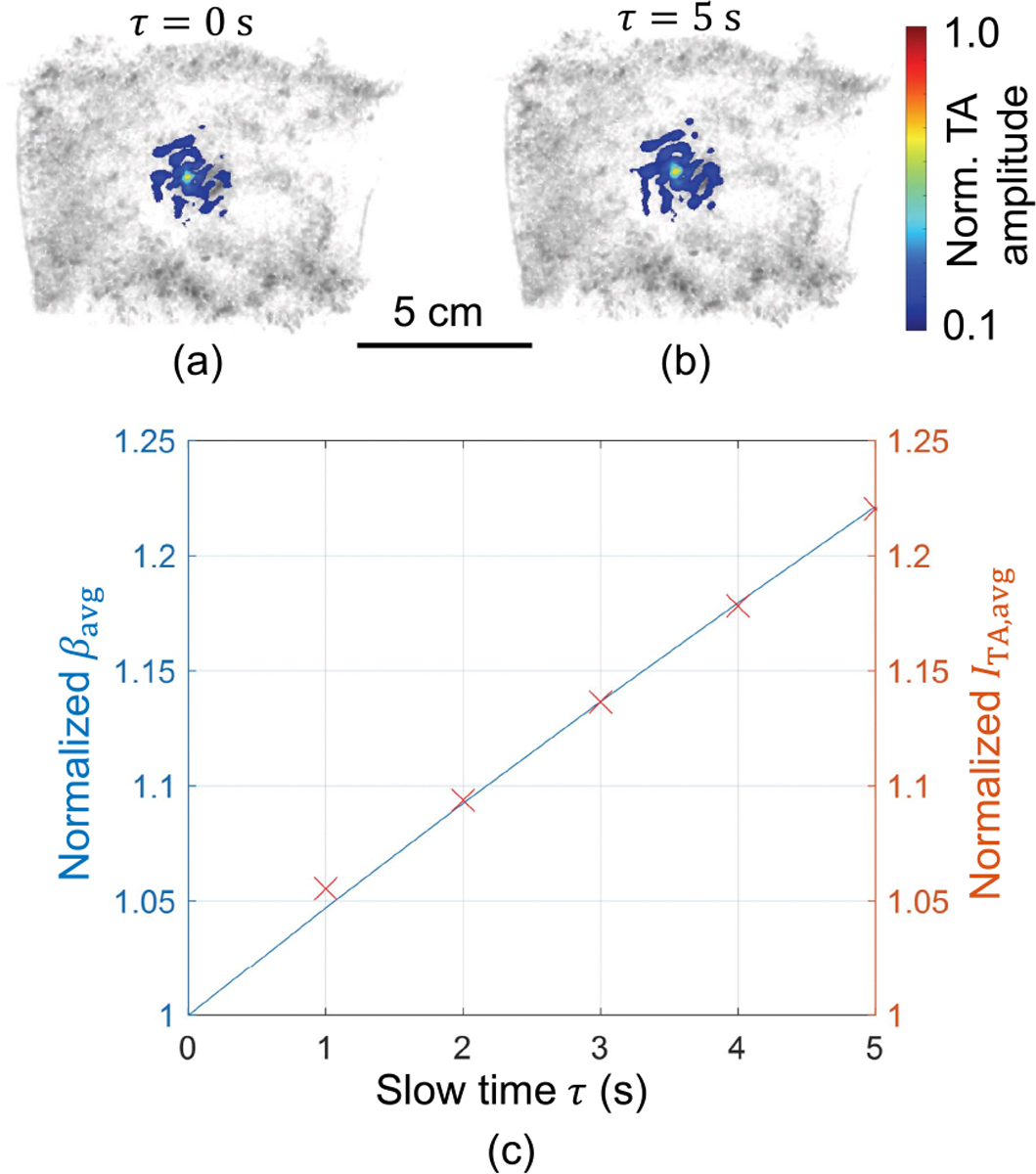
Scaling TA images to a heating function using the temperature-dependent coefficient of thermal expansion. (a) TA image at *τ* = 0 s overlaid on the UST image. (b) TA image at *τ* = 5 s overlaid on the UST image. (c) Example of the average TA amplitude and estimated coefficient of thermal expansion in a 5 mm region around the ablation probe, both normalized to their value at *τ* = 0 s. Both quantities exhibit near-linear increases. We minimized the differences between these values to estimate the scaling factor $\overset{ˆ}{K}$.

We used samples of store-bought fresh bovine liver with an initial temperature of 15–20 °C. MWA was performed for durations of 1, 3, and 5 minutes at 100 W average power. In each case, TA signals were recorded and averaged over 1-s intervals continuously throughout the ablation procedure. Samples were immersed in room temperature water for acoustic coupling to the receiver array. An example TA frame overlaid on a UST image is shown in Fig. 5. We first use the initial increase in TA image amplitude (~15–25% increase over the first 5 s) around the probe to scale to an estimated heating function Q(r,τ). The normalized average TA amplitude and β are shown in Fig. 5(c) for the estimated $\overset{ˆ}{K}$ found from (12). From the estimated scaling factor and TA images, we then approximate the heating function using (11), which is used for thermal modelling.

**FIGURE 6.**
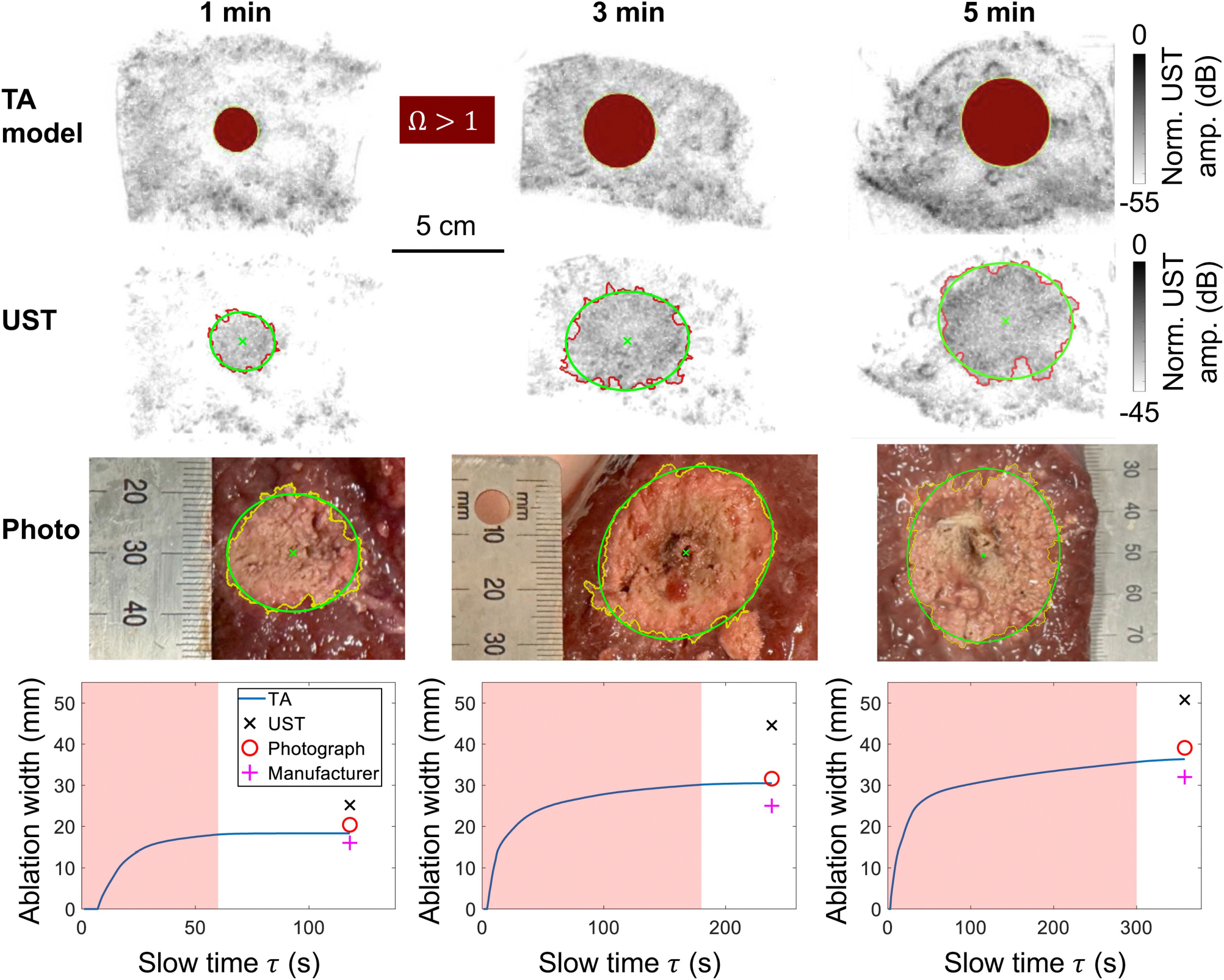
Estimated ablation zones from TA ablation monitoring, UST, and photographs of dissected samples. Columns show three example samples ablated for 1, 3, and 5 minutes. The first row shows the final frame of the estimated ablation zone using TA modelling, where red regions are considered ablated by the Arrhenius damage integral. The second row shows the UST image performed after ablation, where the ablation zone is segmented and fit to an ellipse. The third row shows photographs of the dissected tissue, where the ablation zone is segmented and fit to an ellipse. The final row shows the estimated ablation diameter from TA compared with the other estimates. The red window shows the time when microwave power is applied. We then simulate the thermal model for an additional 60 seconds to estimate the final ablation width.

We performed a UST acquisition approximately one minute after the ablation completion. We then dissected and photographed the liver tissues to assess the ablation diameter. For both UST images and the photographs, we segmented the ablated region and fit an ellipse to it. The ablation zone is estimated as the geometric mean of the major and minor axes of the ellipse. The estimated ablation diameters are compared in Fig. 6. We also compare against the expected ablation widths using the manufacturer’s specifications.

Our results for all six samples are summarized in Table 1. Note that UST consistently overestimates the ablation diameter compared with the photographs. This may be due to the generation of air bubbles and coagulation that increase acoustic scattering outside of the ablation zone, which has been previously observed [26]. We also highlight that for the 3- and 5-minute ablation durations, the ablation width varies by more than 4 mm between the two samples. Whereas the manufacturer specifications cannot discern differences between the two samples, the TA model tracks changes that may be due to differing tissue properties or structures. Despite our simplified model of the coefficient of thermal expansion, we find a lower overall RMS error using TA modelling compared with UST or the manufacturer’s specifications. We expect this could further improve with better characterization of tissue properties over temperature and ablation status. Movies S1-6 show the TA images, estimated temperature distributions, and estimated ablation zones throughout the ablation duration for each sample.

## IV. DISCUSSION

This work introduces a TA imaging framework that models MWA progress and tissue dynamics during the ablation procedure. By modulating the microwave source at MHz-scale frequencies, we generate TA signals whose reconstructions map the spatial distribution of absorbed microwave energy. Treating these TA maps as the heating function term in a thermal diffusion model then provides ongoing estimates of tissue temperature and ablation status throughout the procedure. A brief calibration period, using the first few seconds of data and assuming a temperature-dependent thermal expansion coefficient, links TA image amplitude to absolute power deposition. This calibration implicitly accounts for the acoustic attenuation between the probe and detectors. Since this approach requires no hardware changes to typical MWA generators or antennas, the method is conveniently compatible with existing clinical systems.

This first demonstration highlights both this technique’s promise and areas for refinement. Our implementation infers a 3D heating field from a stack of 2D TA slices, so true volumetric TA acquisition should improve accuracy. Experiments were performed in ex vivo bovine liver lacking perfusion, so future studies could incorporate blood flow, e.g., through the Pennes bioheat formulation [27] or porous-media extensions [28], to capture in vivo heat sinks. Although the technique avoids prior knowledge of tissue permittivity or antenna fields, it still relies on accurate temperature dependences for β and thermal diffusivity. Systematic measurements across tissue types will improve model accuracy and broaden applicability. Steam-generated bubbles could shadow TA signals late in ablation, leading to underestimation of deposited power. A fusion of UST and TA could help account for the acoustic attenuation caused by these bubbles.

The speed of sound in tissue is also expected to change during ablation, from ~1540 m/s at baseline (37 °C) to a maximum of ~1560 m/s at 60 °C [29]. Over a 5 cm diameter ablation zone, this could cause a ~0.6 mm error in our reconstruction of the microwave absorption profile. This would lead to incoherent summation of higher frequency signals recorded with wavelengths less than ~1.2 mm, but the lower frequencies are expected to remain robust to speed of sound changes. Future approaches could perform UST speed of sound estimation throughout the procedure to account for variation during ablation.

To achieve a human-scale field of view, our UST system uses lower frequency (~1 MHz) ultrasound compared to conventional handheld probes (~5–15 MHz). This results in poorer resolution. However, for imaging broader tissue changes like cm-scale tissue necrosis, lower frequencies may be advantageous in visualizing regions deep in the body with reduced acoustic attenuation.

To improve its clinical practicality, skin-coupled detector arrays mounted with an inflatable water bag or conformal pads could collect TA data without immersing the patient in water. Embedding one or more thermocouples in the probe would tighten model calibration and provide a real-time safety check. The hybrid of TA imaging (for energy deposition) and UST (for anatomy) may offer several complementary advantages in the future. UST could run concurrently to provide structural context, measuring temperature-induced speed-of-sound shifts, and identifying macroscopic tissue shrinkage. This may help refine the TA image reconstruction, correct acoustic path aberrations, and improve the fidelity of the thermal model.

In summary, we present a TA-guided thermal-modeling approach that delivers ongoing maps of energy absorption, temperature, and ablation progress during MWA. With continued work on volumetric imaging, perfusion modeling, and bubble mitigation, this technique could enable closed-loop control that adapts power and duration to patient-specific tissue responses, ultimately improving the precision and safety of thermal ablation therapies.

## Supplementary Material

jmw-3612329-mm

This article has supplementary downloadable material available at https://doi.org/10.1109/JMW.2025.3612329, provided by the authors.

## Figures and Tables

**TABLE 1.** Estimated Ablation Widths From the Manufacturer Specifications, UST, TA Modelling, and Photographs of the Dissected Tissues. Errors Are Calculated for Each Modality Relative to the Photographs.

Ablation time (min)	Photo est. (mm)	Manufacturer spec. (mm)	UST est. (mm)	TA est. (mm)	Manufacturer error (%)	UST error (%)	TA error (%)

1	20.4	16	25.2	18.3	−21.6	23.5	−10.2
	19.2		26.3	24.4	−16.5	37.4	27.4

3	31.6	25	44.6	30.5	−20.9	41.1	−3.5
	26.4		44.5	23.3	−5.3	68.4	−11.9

5	32.7	32	44.8	32.8	11.8	56.5	14.6
	39.1		50.8	36.4	−18.2	28.8	−7.0

**Mean error (%)**					**−11.8**	**42.6**	**1.6**
**RMS error (%)**					**16.7**	**45.4**	**14.6**
