# Supplementary material for: Microwave Ablation Monitoring Using Thermoacoustic and Ultrasound Tomography
Source: IEEE J Microw. Author manuscript; Available in PMC 2026 May 1. (PMC13132507; doi:10.1109/jmw.2025.3612329)
Supplement: jmw-3612329-mm [file NIHMS2159843-supplement-jmw-3612329-mm.zip › jmw-3612329-mm/IEEE_JMW_Ablation_Supplementary.pdf]

# Supplementary Materials for

## Microwave ablation monitoring using thermoacoustic and ultrasound tomography

David C. Garrett, Yousuf Aborahama, Jinhua Xu, Geng Ku, and Lihong V. Wang*

*Corresponding author. Email: lvw@caltech.edu

**This PDF file includes:**

Supplementary Text
Figs. S1 to S7
References (1 to 3)

**Other Supplementary Materials for this manuscript include the following:**

Movies S1 to S7
Data S1

**Supplementary Text**

System hardware

An image of a sample before ablation is shown in Fig. S1. The liver sample is immersed in water for acoustic coupling to the receiver array. It is mounted on a plastic support plate held by a single stainless-steel post.

TA amplitude variation during the ablation procedure

Fig. S2 presents the mean TA image amplitude in the tissue immediately surrounding the microwave probe during a 60 s ablation. The initial ~25 % rise over the first ~8–10 s is attributed to the temperature-dependent coefficient of thermal expansion, which we use to scale the TA images to the heating function in eq. (11). The sharp drop in amplitude at ~9 – 15 s is likely due to this region ablating, which agrees with the onset of ablation in our thermal model. After ablation, the microwave absorption drops due to lower water content, resulting in weaker thermoacoustic signals. This is consistent with the rapid drop in the conductivity reported in [2]. The slight increase in amplitude around 35 s could be due to the perfusion of surrounding blood or water perfusing back to the probe, but this would need to be confirmed with further experiments.

Scaling TA images to heating functions

Since our TA images are dimensionless (normalized to the peak amplitude over the ablation time series), we scale them to a heating function through a parameter $K$. We do this by comparing the amplitude rise in the TA images over the first ~5 seconds with the estimated rise in the coefficient of thermal expansion (Fig. S3A) found from the modelled temperature using eq. (9). We choose the scaling factor that minimizes the difference of the normalized TA amplitude and $\beta$ (Fig. S3B).

Comparison with thermometer

We recorded the temperature at 1-second intervals throughout the ablation using a non-conductive fiber thermometer. We extract the location of the thermometer relative to the probe using the UST image before ablation (Fig. S4A). A comparison between the thermometer and TA models is shown in Fig. S5B for a 5-minute ablation. The TA model tracks the initial rise in temperature and the steady high temperature (~100 °C) until the last ~60 seconds, where the thermometer shows a discrete drop then rise in temperature. This may be due to porous regions in the liver sample vaporizing between different compartments, but this phenomenon will require further research. Note that this sample was used for model validation, and it is not included in the six samples we used for ablation width comparison.

Thermal solver

We solve the transient 3D heat equation on a uniform Cartesian grid with uniform spacing of 0.25 mm by an explicit forward-time central-space (FTCS) solver on the GPU (MATLAB gpuArray, single precision). At every time step (10 ms), the specific heat and thermal conductivity of *ex vivo* liver are updated with the empirical fits in eq. (8). The volumetric source term comes from a time-ordered stack of 2D scaled TA images that are axially weighted in the z dimension. The heating function is normalized by the temperature-dependent thermal expansion coefficient ratio to preserve TA calibration to heating function. Cumulative thermal damage is tracked throughout the simulation using the Arrhenius integral. Once $\Omega > 1$, the modeled $\beta$ is switched to a

permanently reduced value ($3 \times 10^{-4}$ °C$^{-1}$) to emulate ablated tissue. During the simulation, we ensure that the FTCS stability condition is met: $dt \leq (\min(\rho C_p))\, dx^2/(6 \max(k))$.

We validate our thermal solver using a 3D instantaneous heat source at $\tau = 0$ s with spatial Gaussian variance $2\gamma = 2(1\text{ mm})^2$, total source energy $E_h = 10$ J, and initial background temperature $T_0 = 20$ °C. We consider a medium with uniform, temperature-invariant properties: $C_p = 3600$ [J · kg$^{-1}$ · K$^{-1}$]; $\rho = 1000$ [kg · m$^{-3}$]; $k = 0.52$ [W · m$^{-1}$ · K$^{-1}$]; $\alpha = k/\rho C_p$.

This results in an initial temperature distribution of:

$$T(\boldsymbol{r}, 0) = T_0 + \frac{E_h}{\rho C_p (4\pi\gamma)^{3/2}} \exp\left[-\frac{|\boldsymbol{r}|^2}{4\gamma}\right]. \tag{1}$$

We insert this distribution in our solver as an initial condition. The theoretical solution is found as [3]:

$$T(\boldsymbol{r}, \tau) = T_0 + \frac{E_h}{\rho C_p [4\pi(\gamma + \alpha\tau)]^{3/2}} \exp\left[-\frac{|\boldsymbol{r}|^2}{4(\gamma + \alpha\tau)}\right], \qquad \tau > 0. \tag{2}$$

We compare the theoretical and numerical results in Fig. S6, where we find close agreement. The temperature ranges from $\sim 20 - 80$ °C, but the maximum error over the entire volume and simulation duration is 0.07 °C.

Ablation widths for all samples

The estimated ablation zones are shown for all samples using UST in Fig. S6 and from photographs in Fig. S8. We first segment the estimated ablation zone boundary using a graph cut in the MATLAB Image Segmentation toolbox. We then fit an ellipse to the segmented boundary and extract the major and minor axis diameters, where the mean ablation width is found as their geometric mean.

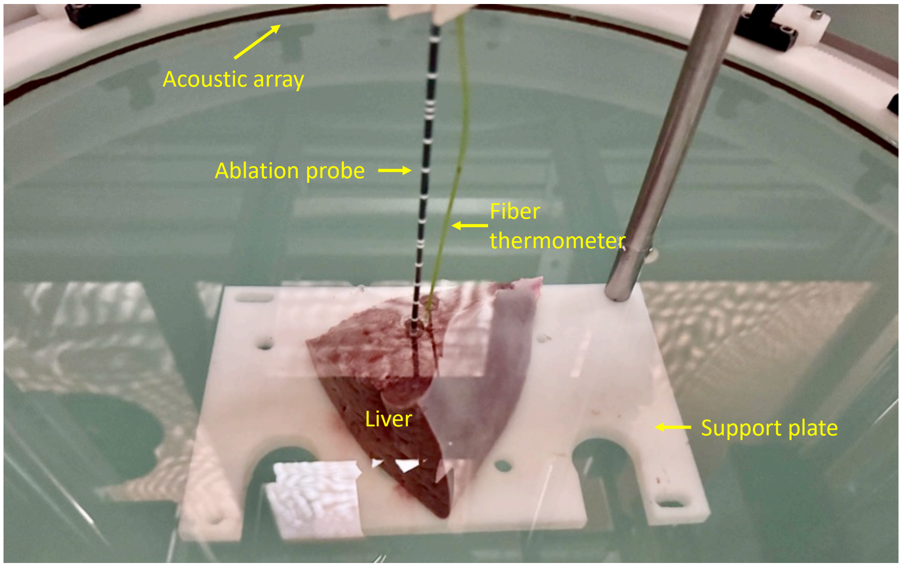


**Fig. S1.**
Photograph of an *ex vivo* bovine liver sample before ablation.

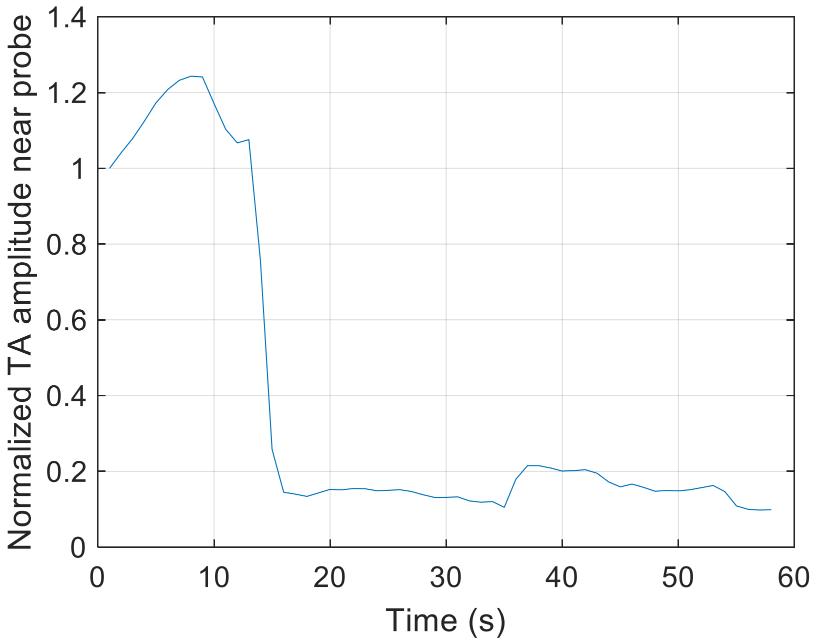


**Fig. S2.**
Example mean TA image amplitude near the probe during a 1-minute ablation.

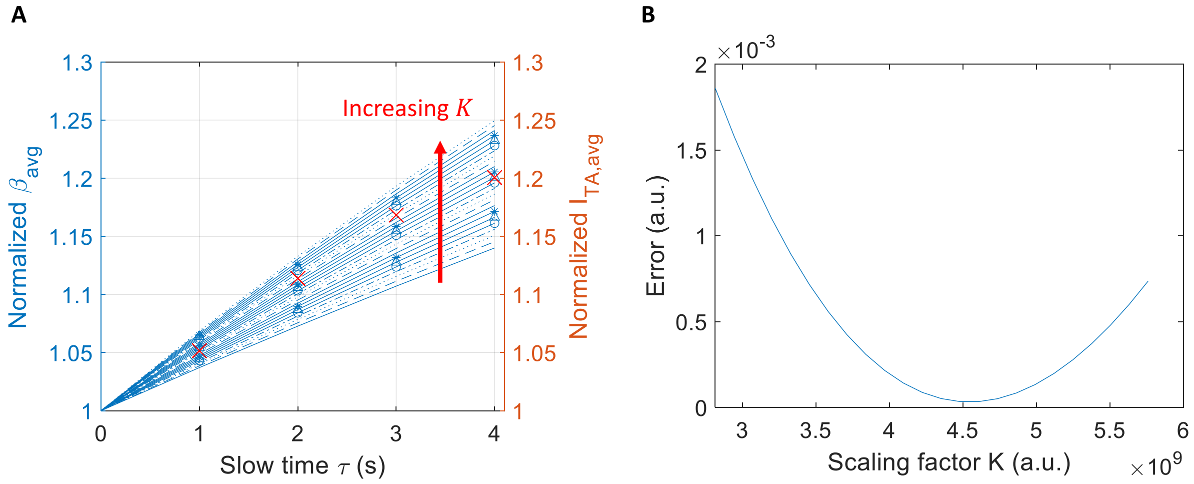


**Fig. S3.**

Determining the scaling factor $K$ between TA images and heating function. (**A**) Example of the normalized TA amplitude rise (red crosses) and normalized $\beta$ from the thermal model (blue curves) for a given scaling factor $K$. (**B**) Error between normalized TA amplitude and $\beta$ for a range of $K$. The optimal $K$ is chosen to minimize this error.

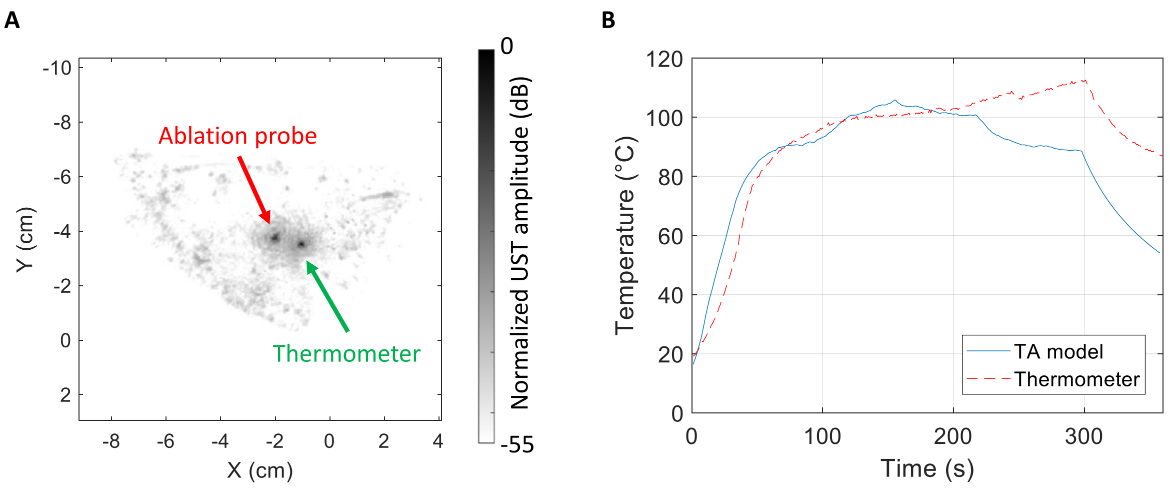


**Fig. S4.**
Extracting fiber thermometer location from UST and comparing estimated temperature against UST model. (**A**) UST image of a liver sample before ablation, showing the location of the ablation probe and fiber thermometer. (**B**) Comparison of the estimated temperature at the thermometer location from the TA model and fiber thermometer.

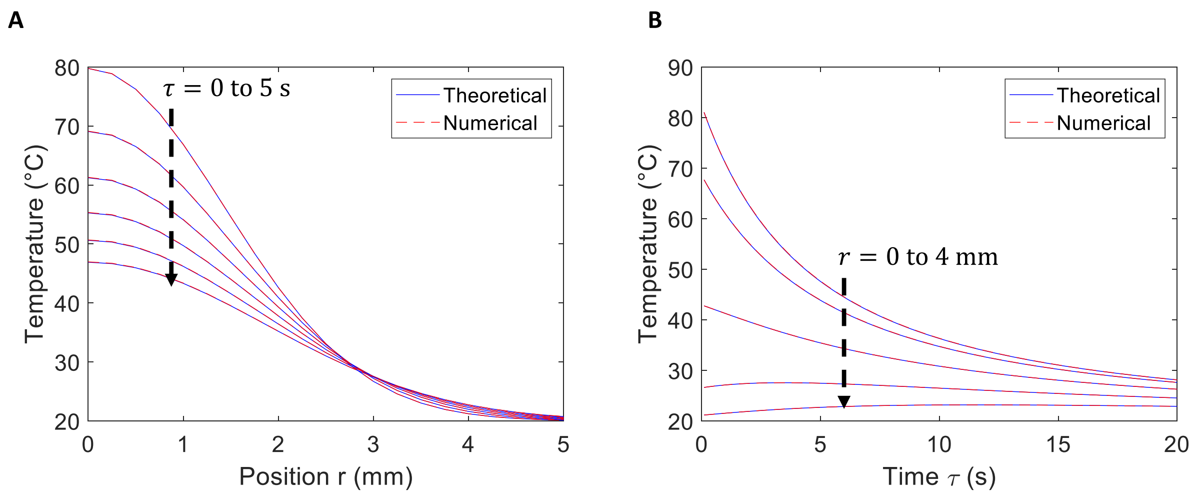


**Fig. S5.**
Validating our thermal solver against a theoretical solution for an instantaneous 3D Gaussian thermal source. (**A**) Theoretical and numerical solutions as a function of position $r$. The six curves are shown for $\tau$ of 0, 1, 2, 3, 4, and 5 s. (**B**) Theoretical and numerical solutions as a function of time $\tau$. The five curves are shown for $r$ of 0, 1, 2, 3, and 4 mm from the source center. The maximum error for the entire simulation volume and duration is 0.07 °C.

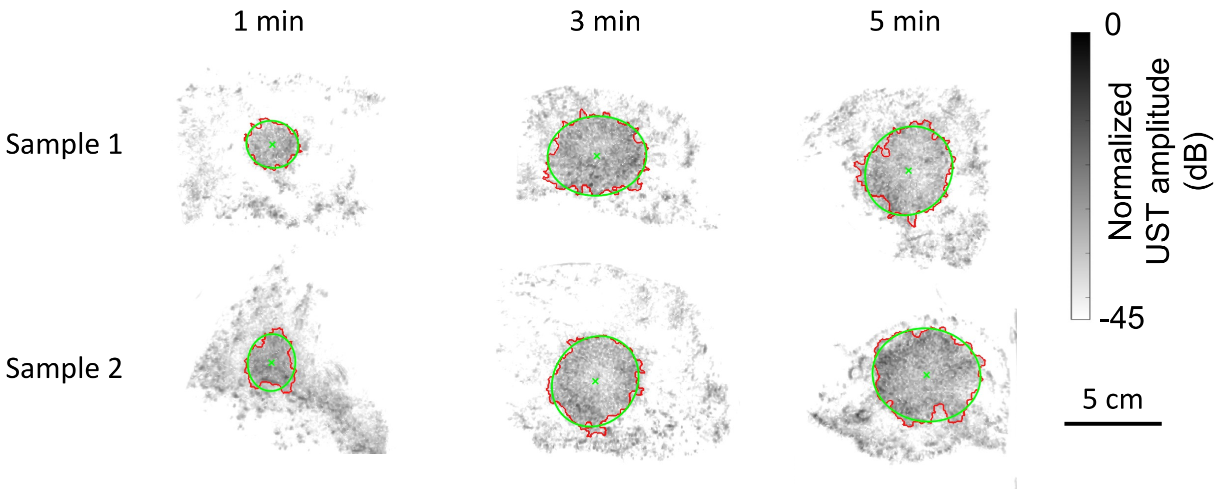


**Fig. S6.**
Estimated ablation zones in the plane normal to the ablation probe from UST. Red contours show the segmented boundaries. Green curves show the fit ellipses to the segmented boundaries.

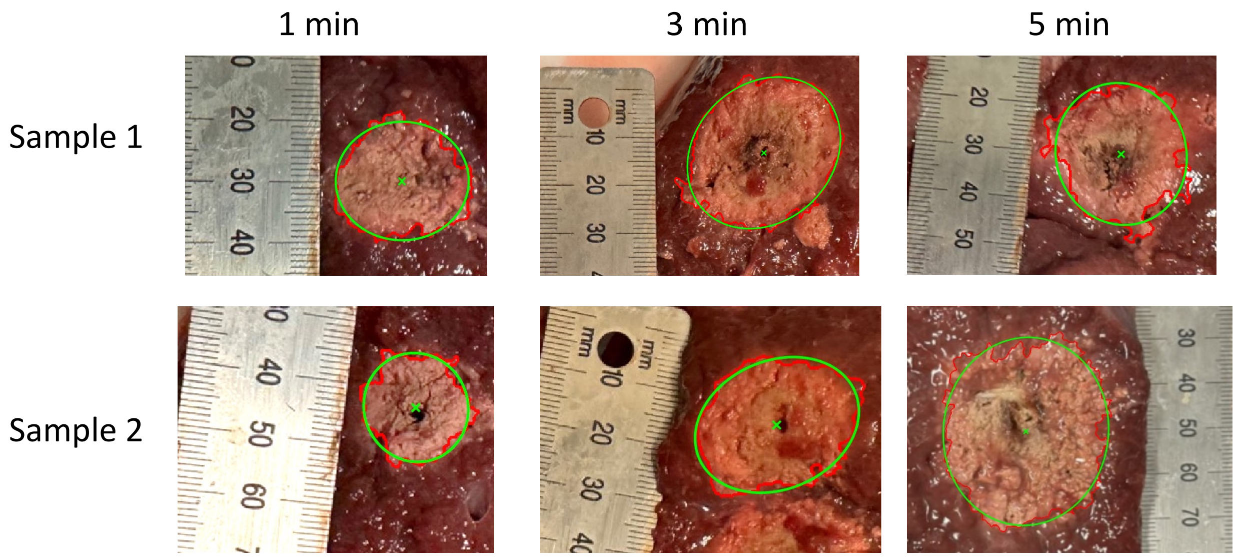


**Fig. S7.**
Estimated ablation zones in the plane normal to the ablation probe from photographs of dissected samples. Red contours show the segmented boundaries. Green curves show the fit ellipses to the segmented boundaries.

**References**

[1] C. L. Brace, "Microwave Ablation Technology: What Every User Should Know," *Curr. Probl. Diagn. Radiol.*, vol. 38, no. 2, pp. 61–67, Mar. 2009, doi: 10.1067/j.cpradiol.2007.08.011.

[2] Z. Ji and C. L. Brace, "Expanded modeling of temperature-dependent dielectric properties for microwave thermal ablation," *Phys. Med. Biol.*, vol. 56, no. 16, pp. 5249–5264, Aug. 2011, doi: 10.1088/0031-9155/56/16/011.

[3] J. Crank, *The Mathematics of Diffusion*. Clarendon Press, 1979.

**Movie S1.**

TA images, estimated temperature, and estimated ablation zone throughout a 1-minute liver ablation at 100 W, overlaid on the UST image before ablation (Sample 1)

**Movie S2.**

TA images, estimated temperature, and estimated ablation zone throughout a 1-minute liver ablation at 100 W, overlaid on the UST image before ablation (Sample 2)

**Movie S3.**

TA images, estimated temperature, and estimated ablation zone throughout a 3-minute liver ablation at 100 W, overlaid on the UST image before ablation (Sample 1)

**Movie S4.**

TA images, estimated temperature, and estimated ablation zone throughout a 3-minute liver ablation at 100 W, overlaid on the UST image before ablation (Sample 2)

**Movie S5.**

TA images, estimated temperature, and estimated ablation zone throughout a 5-minute liver ablation at 100 W, overlaid on the UST image before ablation (Sample 1)

**Movie S6.**

TA images, estimated temperature, and estimated ablation zone throughout a 5-minute liver ablation at 100 W, overlaid on the UST image before ablation (Sample 2)

**Movie S7.**

Example estimate of the 3D ablation zone during a 1-minute ablation at 100 W.

**Data S1. (separate file)**

Reconstructed TA images for all liver samples. The image data `img_all` is in the form $(\tau, x, y)$, where $\tau$ ranges from 60 to 300 s depending on the ablation duration. The 512 $x$ and $y$ positions are stored in `Display.xm` and `Display.ym`, respectively.
